# Professional Recruits

**Published:** 1898-11

**Authors:** Jonathan Taft

**Affiliations:** Cincinnati, O.


ARTICLE VII.

### PROFESSIONAL RECRUITS.

DR. JONATHAN TAFT, CINCINNATI, O.

By referring to the commencement exercises of forty of the principal dental colleges of the country, for the present year, it appears that there have been 1,620 graduates. This will make quite an addition to the ranks of the profession; whether more than enough to supply the demand, is a question about which there is much diversity of opinion.

There are very few who now enter upon the practice of dentistry by any other door than through our colleges, so that it is not a difficult matter to estimate the number of annual increase.

There are now not over 24,000 reputable dentists in practice in this country. As to whether the new recruits are more than sufficient to supply the demand, three or four things may be taken into account.

First. The death rate per thousand. Estimating this at twenty per thousand, per annum, gives 480. It is probably not wide of the mark, to estimate that a number equal to this will withdraw from the practice of the profession per annum this for various reasons. Some on account of age, others for the temptation of some more lucrative business, others from manifest unfitness and still others from a dislike to the profession, Now these influences are constantly operative in the withdrawal of practitioners from the profession.

Another point to be taken into account, is, that the country's population is constantly increasing, and on that account increasing the demand for the service of the dentist; and still further if our country is to receive new additions to its territory, as seems probable now, that will occasion still an increased demand. The probability is from present indications, that in the near future the population of the country will be increased by many millions, by the acquisition of new territory and new people. It is true doubtless that such increase of population will not make a corresponding additional demand for dental service, yet doubtless there will be somewhat of increased demand in the immediate future, and it will increase rapidly as tlme goes on.

Another point to be considered is that the people of our country, and in all civilized countries are becoming more and more intelligent in regard to the value of good dental service. The demand for such services is greater now than ever before and is constantly growing.

Another fact which should not be overlooked is, that there is constantly more or less of demand for dental practitioners in other countries than our own, and this is every year drawing quite a number of our best practitioners from the ranks of the profession. When all these things are taken into account it does not seem probable that in the near future the ranks of the dental profession will be overcrowded.

The people are now demanding better services than ever before, and a great many inefficients are dropping out or being dropped out, because they can not answer the demand for a better quality of service.

Calls are frequently coming for dentists better equipped than those who have occupied places up to the present time, and there are many places that have no dentists within a convenient distance, and some where persons are compelled to go many miles for such services.

Now, having made these statements, it is proper to suggest that a considerable number of the 1,000 newly introduced dentists who are now beginning their career, are poorly equipped and will hardly be able to make a professional success, and this for two or three reasons. Some persist in taking an educational course in dentistry, and who have no natural fitness or adaptation for it. Another class have not the perseverance, energy or industry to accomplish their educational work in an efficient manner. It is here suggested that these facts ought to be a astimulus to all our schools to exercise the utmost discrimination as to the persons they admit to their school, selecting and encouraging those who are well endowed, rejecting those in whom such endowments are absent, and exercising great caution in regard to those who are in an uncertain condition. Such care and discrimination would result in great benefit to the profession and immensely to the welfare of those who require the service of the dentist.—Editorial in *Register*.

